# Comparative Evaluation of the Wear Rate of Reinforced Glass Ionomer Cement and Bulk-Fill Composite Using 3D Scan in Extracted Teeth and Its Association With Clinical Wear Rate in Class I and II Restorations: A Randomized Clinical Trial

**DOI:** 10.7759/cureus.101967

**Published:** 2026-01-21

**Authors:** Kiranmayi Govula, Kowmudi Maddineni, Pavan Kumar, Swapna Sannapureddy, Sunil Kumar Chinni, Lavanya Anumula

**Affiliations:** 1 Conservative Dentistry and Endodontics, Narayana Dental College and Hospital, Nellore, IND; 2 Conservative Dentistry and Endodontics, Kamineni Institute of Dental Sciences, Nalgonda, IND

**Keywords:** 3d volumetric assessment, bulk-fill composite, chewing simulator, clinical correlation, direct restorations, reinforced glass ionomer cement, wear analysis

## Abstract

Background: Reinforced glass ionomer cements (GICs) have gained attention as potential durable alternatives to resin composites for posterior restorations, owing to their improved mechanical properties and fluoride release. However, limited standardized protocols exist to assess their long-term wear resistance and clinical performance, particularly in comparison with bulk-fill resin composites under variable patient-related factors.

Aim: This study aimed to develop a standardized protocol to evaluate the suitability of reinforced GIC as a durable permanent direct restorative material in Class I and Class II cavities compared with bulk-fill resin composites and to correlate in vitro findings with clinical wear behavior.

Materials and methods: The study consisted of two phases: an in vitro experimental phase followed by a correlational clinical trial. In the in vitro phase, 40 extracted human molar teeth were collected and divided according to cavity type: Group 1 (Class I cavities; n = 20), Group 2 (Class II cavities; n = 20), and subgroups a (reinforced GIC; n = 10) and b (bulk-fill composite; n = 10). The restored samples were stored in distilled water for 24 hours and subjected to simulated mastication cycles equivalent to six and 12 months of clinical function using a chewing simulator. Volumetric wear was determined using the Geomagic software (3D Systems, Rock Hill, SC, USA). In the clinical phase, restorations made of the same materials and with the same cavity configurations were placed in patients and monitored periodically for three-dimensional wear and volumetric changes.

Results: The wear was assessed using the Geomagic Control X (3D Systems, Rock Hill, SC, USA) and WearCompare software on replica models, and restorations were analyzed using the revised Fédération Dentaire Internationale (FDI) criteria.

Conclusion: The standardized in vitro-in vivo protocol provides a reliable framework to assess the durability and clinical relevance of reinforced GICs compared with bulk-fill composites in posterior restorations, offering insight into their long-term functional behavior under patient-related influences.

## Introduction

Human dentition is subjected to various insults, including physical, mechanical, chemical, and biological. Mechanical insults, such as wear, abrasion, and attrition, are of significant concern, particularly in the posterior teeth, as they are subjected to the forces of chewing [1,2]. Most of the posterior teeth require restorations because of carious or non-carious lesions, developmental defects in enamel and dentin, and failed restorations [3]. Among posterior restorations, Class II restorations are the most common, as they involve both the occlusal and proximal surfaces of teeth. Restorative materials used to restore these areas are subjected to severe occlusal and proximal wear.

The mechanism of wear in restorations is complex. It can be described as a multifactorial process involving a complex interplay among many host factors [4,5], in which the influence of patient factors may be primary. In addition to age and gender, other factors, such as the increase in vertical dimension of occlusion (VDO), bite forces, and etiological factors (including mechanical and chemical-related factors), may also be important in the wear process [6]. Researchers hypothesized that mechanical challenges would lead to greater wear of composites over time compared to chemical difficulties and that a larger increase in VDO and high bite force would result in increased wear [7]. The direct restorative materials used to restore Class I and Class II restorations, along with natural tooth substance, exhibit good wear resistance.

Restorative materials should be tested for their wear resistance in the laboratory and be evaluated in the clinical scenario [8]. To date, no such violated methods exist, as wear is a complex dynamic process that can hardly be assessed by clinical examination alone; therefore, objective monitoring methods are required that can directly compare follow-up data to baseline information on wear using plaster models or three-dimensional (3D) scans. Quantitative information on wear is needed. Intraoral 3D scans or information or scans of replicas after impression taking should be considered as the method of choice [9].

As there is a lack of well-validated laboratory methods for predicting the clinical wear performance of direct restorative materials, particularly glass ionomer and compomers, this study aimed to correlate in vitro test procedures with the clinical evaluation of the wear performance of glass ionomer cement (GIC) and composite restorative materials using a 3D intraoral scanner.

The proposed research results address the gap in understanding the wear of direct restorative materials, thereby benefiting students and clinicians by providing knowledge of the properties, selection, and applications of these materials. It helps evaluate whether reinforced GIC is comparable to resin composites for restoring occlusal cavities in areas under constant force-bearing. Hence, the study's results can enhance the selection of direct restorative materials for restoring Class I and II cavities, which are the most common and crucial.

Henceforth, the aim of the in vitro study was to evaluate and compare the wear rate of reinforced GIC and bulk-fill composite material when restored in Class I and II cavities. In addition, the aim of the clinical study was to develop a standardized in vitro test protocol to determine the suitability of reinforced GIC as a durable permanent direct restorative material in Class I and Class II cavities compared to bulk-fill resin composite. As part of this research, the study compared the clinical performance of reinforced GIC and bulk-fill resin composite, using the revised Fédération Dentaire Internationale (FDI) criteria and 3D laser scan methods for wear analysis in parallel. Furthermore, the study aimed to investigate the wear behavior of reinforced GIC and bulk-fill composite resin restorations in relation to the revised FDI criteria.

## Materials and methods

Methodology

Preliminary Research

Laboratory investigations: A total of 40 extracted human molar teeth were collected and stored in distilled water. Based on cavity configuration, the teeth were divided into two groups: Group 1, which included Class I designs, and Group 2, which included Class II designs. In Group 1, standard Class 1 cavities (2 × 2 × 2 mm³) were prepared in 20 extracted sound human molars. Depending on the type of restorative material, it is subdivided into two groups (n = 10): 1a, restored with GlasIonomer FX Ultra (Shofu Dental India Pvt. Ltd./Shofu Inc., New Delhi, India), and 1b, restored with Tetric EvoCeram (Ivoclar Vivadent AG, Schaan, Liechtenstein) composite. Class II cavities (2 × 2 × 1 × 1 mm^3^) were prepared in 20 extracted sound human molar teeth in Group 2. Depending on the type of restorative material, it was subdivided into two groups (n = 10), i.e., Group 2a (GlasIonomer FX Ultra) and Group 2b (Tetric EvoCeram composite). After restorations, the teeth were stored in distilled water for 24 hours. All the samples were subjected to a chewing simulator for six and 12 months of chewing cycles. After the chewing simulation terminated, the abrasion volume was calculated from a 3D scan and superimposed in the Geomagic software (3D Systems, Rock Hill, SC, USA) to determine the number of cycles achieved. 3D scan images of the restorations were recorded both before and after cyclic testing. After the tooth was removed from the testing rig, the abrasion volume was determined as the difference in volume between the reconstructed images. The pre- and post-scans of the teeth were superimposed as volume models based on geometric features to calculate volumetric differences. Quantitative analysis of occlusal wear using a 3D laser scan was performed to assess vertical and total volume loss across the entire restoration surface. 

Chewing simulator parameters: The following parameters were used: applied load (specify, e.g., 50 N), number of cycles per interval (e.g., 100,000 cycles), frequency (e.g., 1.2 Hz), antagonist material (e.g., steatite/stainless steel/enamel cusp), motion pattern (vertical loading combined with horizontal sliding movement), environment (simulated oral conditions using artificial saliva), and abrasive medium (specify if used, e.g., slurry/none). These parameters were selected to simulate functional occlusal loading conditions relevant to posterior restorations.

Results

3D laser scan quantitative analysis: Occlusal wear was quantified based on vertical height loss and volumetric loss across the restoration surface. Table 1 presents the cumulative mean vertical loss for the two materials over six months and one year. Between six months and one year, both the materials exhibited a running-in wear pattern, indicating progressive surface adaptation under masticatory forces. After one year, the mean vertical wear was 64 ± 26 μm in the GlasIonomer FX Ultra group and 75 ± 27 μm in the Tetric EvoCeram group. A statistically significant difference was detected between the two groups (p < 0.05; paired t-test). However, no significant difference in vertical wear was observed between Class I and Class II restorations (p = 0.24), suggesting that cavity configuration did not substantially influence vertical wear in this study.

**Table 1:** Cumulative mean of the vertical loss A paired t-test was performed, and a p-value of <0.05 was considered statistically significant. * means statistically significant.

Groups		Intervals	Mean	Std. deviation	t-value	P-value
Group 1	6 months	Initial alignment	-0.0063	0.00452	19.89	0.000*
		3D	-0.1086	0.01206		
	12 months	Initial alignment	-0.0125	0.00206	6.268	0.008*
		3D	-0.1126	0.03202		
Group 2	6 months	Initial alignment	-0.0026	0.00717	13.302	0.001*
		3D	-0.0898	0.01027		
	12 months	Initial alignment	-0.0099	0.00299	6.111	0.009*
		3D	-0.1178	0.03266		

Table 2 presents a comparison of initial and 3D alignment changes between the two groups at six and 12 months, revealing no statistically significant differences (p > 0.05). Although Group 1 showed slightly greater changes in initial alignment at 12 months compared to Group 2, this difference did not reach statistical significance (p = 0.060). Similarly, for 3D alignment, both groups demonstrated comparable changes at six and 12 months, with no significant intergroup variation. These findings indicate that although both GIC and composite restorations undergo measurable occlusal wear over time, composites demonstrated superior wear resistance compared with GIC after one year of clinical service. The absence of significant differences between Class I and Class II restorations suggests that the type of cavity configuration may play a limited role in influencing wear behavior. 

**Table 2:** Intergroup comparison An independent t-test was used, with a p-value of <0.05 considered statistically significant. NS: not significant

Groups		Intervals	Mean	Std. deviation	t-value	P-value
Initial alignment	6 months	Group 1	-0.0063	0.00452	-0.850	0.428 (NS)
		Group 2	-0.0026	0.00717		
	12 months	Group 1	-0.1086	0.01206	-2.367	0.06 (NS)
		Group 2	-0.0898	0.01027		
3D alignment	6 months	Group 1	-0.0125	0.00206	-1.417	0.206 (NS)
		Group 2	-0.0099	0.00299		
	12 months	Group 1	-0.1126	0.03202	0.227	0.828 (NS)
		Group 2	-0.1178	0.03266		

The results suggest that a clinical perspective is necessary; therefore, a clinical trial was conducted to test both treatment approaches, which demonstrated comparable improvements in initial and 3D alignment over six and 12 months, with no statistically significant differences. From a laboratory perspective, the time-dependent variations observed underscore the need for extended simulation models that better reflect clinical aging and environmental stress. Hence, these findings suggest that either protocol can be used effectively to achieve and maintain alignment, as neither showed clear superiority in clinical outcomes during the study period.

Research question: Can GIC with enhanced properties withstand the wear when restored as Class I and II restorations compared to bulk-fill composite?

PICOS

Population/problem: The population comprised patients presenting with Class I and Class II carious lesions in mandibular molar teeth.

Intervention: The intervention involved restoration using reinforced conventional GIC (GlasIonomer FX Ultra).

Comparison: The comparison involved restoration using a bulk-fill resin composite (Tetric EvoCeram).

Primary outcomes: Clinical performance was assessed using the revised FDI criteria, including postoperative hypersensitivity, secondary caries, gross fracture, color match, cavo-surface marginal discoloration, marginal integrity, and proximal contact.

Secondary outcome: Quantitative wear assessment was performed using 3D scanning analysis.

Null hypothesis: It was hypothesized that (1) wear of composite restoration on Class I and Class II would be similar and 2) mechanical wear of composite restorations over time compared to GIC and composite wear would be the same.

Study population: Following the approval of the study protocol by the institutional ethics committee, subjects were recruited from patients seeking treatment in the outpatient department of the Department of Conservative Dentistry and Endodontics. These patients were included after screening.

Sample size: The same operator performed all restorations to ensure consistency. Forty subjects diagnosed with at least one Class I and II carious lesion, selected from the outpatient department, were included in the study. An a priori sample size calculation was performed for the clinical component of the study, considering wear (quantitative outcome) as the primary endpoint and FDI criteria scores (categorical outcomes) as secondary endpoints. For the quantitative wear analysis, the sample size was calculated based on detecting a clinically meaningful difference in mean wear values between the reinforced GIC and bulk-fill composite groups. Based on data from previously published studies evaluating posterior restorative wear, a mean difference of 20-25 µm, with a pooled standard deviation of 25 µm, was considered clinically relevant. Using a two-tailed test, a significance level (α) of 0.05, and a statistical power of 80%, the minimum required sample size was calculated to be 20 restorations per group. To compensate for potential dropouts and loss to follow-up during the 12-month clinical evaluation, the sample size was increased by approximately 10-15%, resulting in a final target sample of 20-25 restorations per group. This sample size was considered adequate to detect statistically and clinically relevant differences in wear outcomes. The in vitro phase was designed independently to provide mechanistic insight into material wear behavior and was not used to determine the clinical sample size.

Ethical approval: The study was selected for the Faculty Research Grant 2023 (4/FRG/2023) by Dr. NTR University of Health Sciences, Vijayawada. The study got approval from the Institutional Ethics Committee of Narayana Dental College and Hospital (approval number: IEC/NDCH/2023/AUG-SEPT/P‑88). All the volunteers received complete dental treatment in the Department of Conservative Dentistry and Endodontics at Narayana Dental College and Hospital, Nellore.

Trial design, settings, and data collection location: The trial was a double-blinded, randomized, and equally allocated clinical trial. The study was conducted in the Department of Conservative Dentistry and Endodontics at Narayana Dental College and Hospital, Nellore, from March 2024 to August 2025, including follow-up visits.

Registration: The clinical trial was registered under Clinical Trials Registry - India (CTRI) (CTRI/2024/04/065017).

Recruitment: Subjects were recruited as they sought treatment in the tertiary hospital, and subjects were recruited in the order in which they reported for screening sessions, resulting in a convenience sample, as explained in the consort diagram (Appendix A).

Eligibility

Inclusion criteria: Individuals aged 18-55 with an occluding tooth, good oral hygiene, and at least two carious lesions rated with an International Caries Detection and Assessment System (ICDAS) score of 4 or 5 were included in the study.

Exclusion criteria: Patients with poor periodontal status, complicated medical history, potential behavioral problems, and carious lesions having wear facets, bruxism, and a deep bite were excluded from the study.

**Figure 1:**
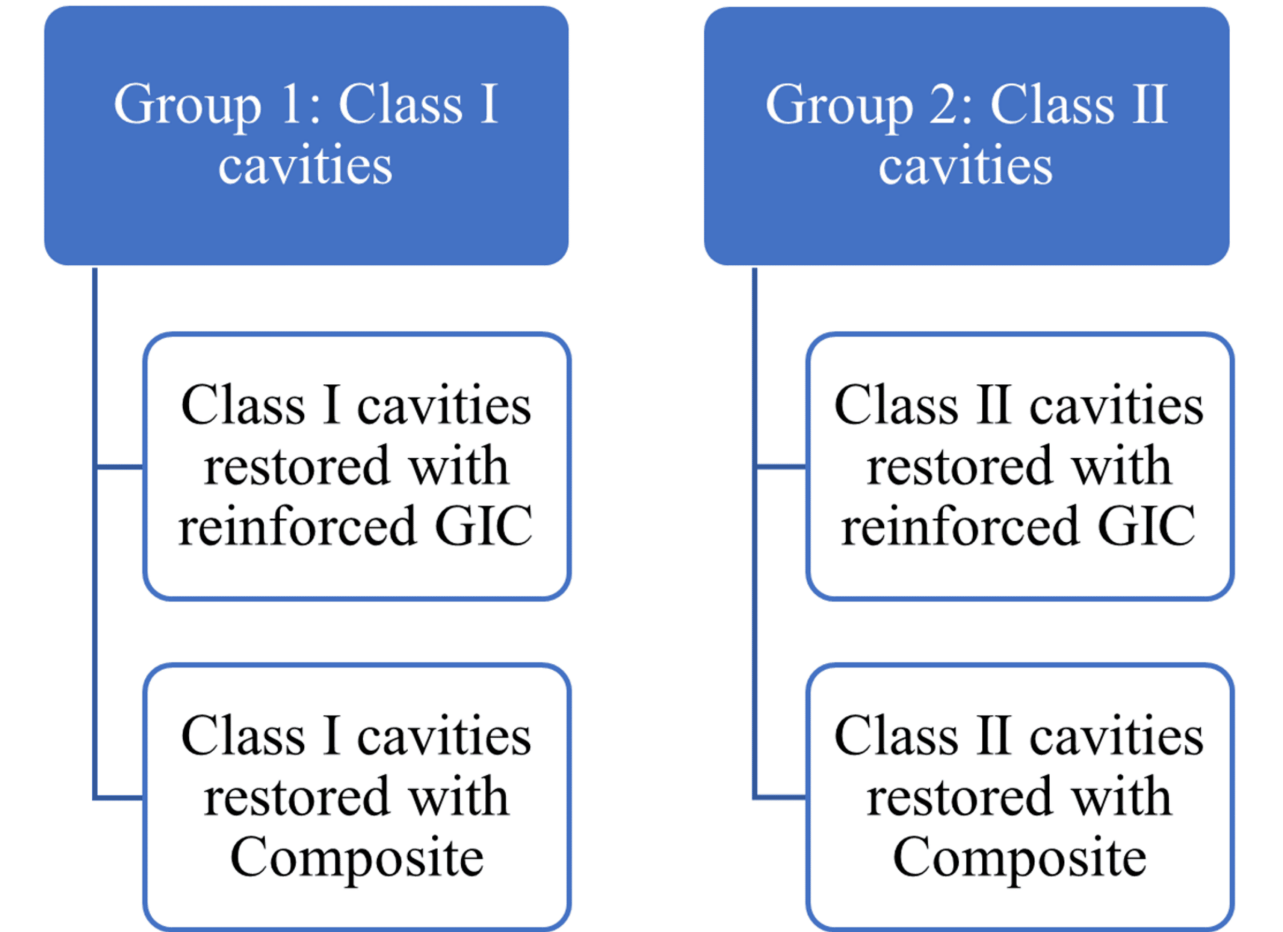
Randomization into two groups based on the restorative material used and subgroups based on the type of cavity preparation GIC: glass ionomer cement

Based on cavity configuration, the teeth were divided into two groups, each comprising 20 teeth: Group 1, with a Class I design, and Group 2, with a Class II design. Again, each group is subdivided into two subgroups, depending on the restorative material used: subgroup a (reinforced GIC) and subgroup b (composite) (Figure 1).

Participants were allotted into subgroups a and b based on the type of material used: in case of an odd number, GIC restoration materials, and in case of an even number, composite restoration materials.

Randomization Sequence Generation and Allocation Concealment

Block randomization was followed to ensure randomness. Randomization was followed separately for Class I and II cavities. A block of four numbers was chosen from the random table. Before restoration, one number was selected from a block of four. If the number is odd, the repair was done with the reinforced GIC Ultra FX. If the number was even, the restoration was done with Tetric EvoCeram composite bulk-fill posterior restorative material for the first tooth. The first tooth is counted based on its position in FDI notation.

Interventions: The same operator performed all restorations to ensure consistency. Before restorative procedures, peri-apical radiographs of the teeth to be treated were taken. All the patients were informed about the study's aim, nature, and design. Before the commencement of the study, written informed consent forms were obtained from the patients.

Forty subjects registered under outpatient care and diagnosed with at least one ICDAS code 4-5, including Class I and Class II carious lesions selected from the outpatient department, were included in the study. If the caries was deep, sensibility tests were also performed. The wear of the restorative material was assessed at baseline, three months, six months, and one year. FDI criteria were taken into the study [10].

Restoration Procedure

Class I cavity preparation (Group 1): After isolating the tooth with a rubber dam or cotton rolls, the caries was excavated with a round tungsten carbide bur. In subgroup 1a, after cavity preparation (n = 10), the cavity was dried, and GIC Ultra FX was mixed according to the manufacturer's instructions. It was then placed in the cavity, followed by finishing and polishing. In subgroup 1b, the cavities (n = 10) with a depth of <3 mm were not recruited for composite placement. Instead, each cavity was acid-etched with 37% phosphoric acid for 15 seconds, rinsed for one minute, and air-dried. A dentin bonding agent was applied, air‑dried for five seconds, and light‑cured for 20 seconds. Teeth were restored with Tetric EvoCeram bulk-fill posterior restorative material based on the randomization and were light‑cured, followed by finishing and polishing.

Class II cavity preparation (Group 2): After isolating the tooth with a rubber dam or cotton rolls, the caries were excavated with a round tungsten carbide bur. For subgroup 2a, after cavity preparation, it was dried and isolated using a sectional matrix. GIC Ultra FX was mixed according to the manufacturer's instructions and placed in the cavity. For subgroup 2b, the cavities with <3 mm depth were not recruited in the study. After applying the sectional matrix, the cavity was acid‑etched with 37% phosphoric acid for 15 seconds, rinsed with water for one minute, and air‑dried. A dentin bonding agent was applied, air‑dried for five seconds, and light‑cured for 20 seconds. Teeth were restored with Tetric EvoCeram and light‑cured, followed by finishing and polishing.

To accurately measure in vivo wear of restorations, it is necessary to make replicas of the restoration and the surrounding tooth before and after wear over three, six, and 12 months and to compare their wear patterns. Impressions were taken at baseline after restorations and at follow-up visits using polyvinyl siloxane (PVS) impression material (GC Flexceed Putty and Light Body, GC Corporation, Hyderabad, India), which was selected for its superior dimensional stability, fine detail reproduction, and minimal polymerization shrinkage. It was mixed according to the manufacturer's instructions. The obtained impressions were immediately inspected for voids or surface imperfections. Only defect-free impressions were used for model fabrication, and the study models were poured using Type IV dental stone.

The models were scanned three-dimensionally, and these scans are saved as standard tessellation language (STL) files. The STL files from the baseline and follow-up scans are imported into Geomagic Control X (3D Systems, Rock Hill, SC, USA) [11]. The baseline scans serve as a reference for each type of restorative material. In contrast, the relevant follow-up scan is imported as the measured data for comparison. The initial alignment and best-fit algorithm were used to superimpose in the software [12,13]. The surface details of each restoration were individually segmented in the baseline scan and compared with its follow-up scan using the 3D comparison tool in the metrology software program (Geomagic; 3D Systems, Inc., Rock Hill, SC, USA). The software generated a color scheme spanning 500 μm to visualize discrepancies, with the measured values displayed in millimeters. Yellow-to-red areas indicate volume increase, while light-to-dark blue areas indicate volume loss. To evaluate the results of tooth wear, the values for maximum occlusal height loss (maximum point of loss recorded over the occlusal surface) and mean profile loss (average surface loss over the occlusal surface) were used.

Volumetric changes between baseline and follow-up were visualized using a 3D color deviation map, following a model analysis performed with the Geomagic Control X (3D Systems) and WearCompare software. The baseline (reference) and follow-up period (test) STL files were superimposed using best-fit alignment to assess surface deviations. The color scale (in microns) indicates the magnitude and direction of surface change, where green represents minimal deviation, blue shades indicate areas of material gain or positive deviation, and red to yellow regions correspond to material loss or negative deviation. The data acquired from the quantitative analysis were statistically analyzed.

Evaluation criteria: A standardized scoring form was used by each evaluator at each recall, ensuring that evaluators remained blinded to earlier evaluations during follow-up. If a disagreement occurs between the examiners, the final decision is made by consensus. The restorations were evaluated using the revised World Federation criteria (FDI) (fracture of material and retention, marginal adaptation and integrity, secondary caries, cavo-surface margin discoloration, postoperative sensitivity, gross fracture, proximal contacts, and color match). The restorations were evaluated at baseline (immediately after restoration) and at three, six, and 12 months. All the restorations received the following scores: clinically acceptable (scores 1-3), repairable (score 4), and replacement (score 5). 

Clinical follow-up and recall management: Clinical follow-up evaluations were scheduled at predefined intervals (baseline, six months, and 12 months). Recall compliance was ensured through appointment reminders via telephone and electronic communication, and follow-up visits were coordinated at the time of initial treatment completion. Patients who missed scheduled visits were contacted and rescheduled within a predefined recall window. Dropouts and missed visits were documented, and reasons for attrition were recorded when available. Analyses were performed using available-case data, with all restorations that completed each follow-up interval included in the corresponding analysis. No replacement of dropouts was undertaken. To ensure procedural consistency, baseline occlusal adjustment was performed immediately after restoration placement using articulating paper, and restorations were refined until no premature contacts were detected. Polishing procedures were standardized using the same polishing system and sequence for all restorations at baseline. No re-polishing or occlusal refinement was performed during follow-up visits unless clinically indicated for patient comfort or restoration integrity; such interventions were recorded.

Statistical analysis: Statistical analysis was performed using IBM SPSS Statistics for Windows, V. 26.0 (IBM Corp., Armonk, NY, USA). Descriptive statistics for quantitative variables were expressed as mean ± standard deviation, while categorical variables were presented as frequencies and percentages.

Normality assessment: Normality of quantitative wear data was assessed using the Shapiro-Wilk test, which is appropriate for small to moderate sample sizes. Data with a p-value of >0.05 were considered normally distributed. Data with a p-value of ≤0.05 were considered non-normally distributed (Table 3).

**Table 3:** Normality testing of quantitative wear variables (Shapiro-Wilk test) GIC: glass ionomer cement

Variable	Group	Time interval	Shapiro-Wilk statistic	P-value	Distribution
Linear wear (µm)	Reinforced GIC	Baseline-6 months	0.96	0.24	Normal
Linear wear (µm)	Reinforced GIC	6-12 months	0.95	0.10	Normal
Linear wear (µm)	Bulk-fill composite	Baseline-6 months	0.97	0.31	Normal
Linear wear (µm)	Bulk-fill composite	6-12 months	0.94	0.08	Normal
Volumetric wear (mm³)	Reinforced GIC	12 months	0.92	0.03	Non-normal
Volumetric wear (mm³)	Bulk-fill composite	12 months	0.91	0.02	Non-normal

Inferential analysis: Between-group comparisons of quantitative wear values were performed using the independent samples t-test for normally distributed data or the Mann-Whitney U test for non-normal data. Within-group comparisons across time intervals were analyzed using repeated-measures analysis of variance (ANOVA) (normal data) or the Friedman test (non-normal data). Categorical clinical outcomes assessed using the revised FDI criteria were analyzed using the chi-squared test or Fisher's exact test, as appropriate. Repeated categorical assessments were treated as paired observations, and interpretation accounted for within-subject correlation. The level of statistical significance was set at p<0.05 for all analyses. 

## Results

3D wear analysis software settings

To improve transparency and reproducibility, a tabulated summary of Geomagic Control X and WearCompare settings has been added to the manuscript. This includes the following: alignment method (best-fit alignment), reference area selection (non-wear or stable peripheral regions), tolerance thresholds (e.g., ±0.05 mm), color scale limits (fixed scale with upper and lower deviation limits), deviation computation (point-to-surface distance mapping), wear outcome extraction (total volumetric change (mm³)), mean positive and negative deviation (µm), and maximum surface deviation values. All STL files were processed using identical software settings to ensure consistency across samples and time points.

The restorations were evaluated independently by two calibrated examiners using the revised FDI criteria. Cohen's kappa statistic was used to test the inter-examiner agreement. In all statistical tests, the significance level was 0.05%. In the event of disagreement between the two examiners, consensus was reached through discussion. A third examiner was consulted when consensus could not be reached. Examiner agreement was high, with a kappa coefficient of 0.82 (Table 4). Minor discrepancies were observed in the esthetic evaluation, which were resolved by consensus.

**Table 4:** Cohen's kappa statistic was used to test the inter-examiner agreement FDI: Fédération Dentaire Internationale

Revised FDI criteria	Examiner 1	Examiner 2
Fracture of material and retention	0.95	0.97
Marginal adaptation and integrity	0.93	0.97
Secondary caries	0.83	0.88
Cavo-surface marginal discoloration	0.82	0.86
Postoperative sensitivity	1.00	1.00
Gross fracture	0.84	0.89
Proximal contact	0.96	0.91
Color match	0.89	0.91

Revised FDI criteria

Fracture Resistance and Retention

Both GIC and composite resins are reliable for Class I and Class II restorations, providing durability of up to six months. At 12 months, a slight decline in performance was observed, more evident in Class II cavities and in GIC, though this was not statistically significant. Over 12 months, both GIC and composites demonstrated good clinical success, with no significant differences between the groups.

Marginal Adaptation and Integrity

At baseline, three months, and six months, both Class I and II GIC and composite restorations demonstrated 100% retention, with no evidence of failure or dislodgement (p = 1.000). At 12 months, minor changes were observed across both restorative groups. In Class I cavities, GIC restorations showed 10% alterations, while composite restorations showed 20% alterations. For Class II restorations, GIC exhibited slightly lower performance (30% scored 2) compared to composite restorations (20% scored 2). However, these variations were not statistically significant (p = 0.741).

Secondary Caries

At baseline, three months, and six months, Class I and Class II restorations with both GIC and composite received a score of 1 (100%), indicating excellent clinical performance with no detectable deterioration. At the 12-month follow-up, minor changes were observed in Class I composite restorations and in Class II GIC and composite restorations. Statistical analysis revealed no significant differences among the groups at any interval (p > 0.05) (Appendix B).

Cavo-Surface Margin Discoloration

At baseline, three months, and six months, all Class I and Class II restorations with both GIC and composite scored 1, reflecting excellent clinical performance without any deterioration. By the 12-month follow-up, however, slight changes were observed: 10% of Class I GIC restorations, 20% of Class I composite restorations, 40% of Class II GIC restorations, and 30% of Class II composite restorations. Importantly, no restoration was rated below 2 at any interval. Statistical analysis revealed no significant differences among the groups at any time point (p > 0.05) (Appendix C).

Postoperative Sensitivity

At baseline, three months, and six months, all Class I and Class II restorations with both GIC and composite were scored as 1 (100%), indicating excellent performance with no detectable deterioration. At the 12-month follow-up, minor changes were observed: 10% of Class I GIC, 20% of Class I composite, 20% of Class II GIC, and 30% of Class II composite restorations shifted to score 2. None of the restorations was rated below 2 at any point. Statistical analysis revealed no significant differences among the groups at any time interval (p > 0.05) (Appendix D).

Gross Fracture

At baseline, three months, and six months, all Class I and Class II restorations with both GIC and composite showed a score of 1, indicating excellent clinical performance with no deterioration. At the 12-month follow-up, a slight decline was noted: 10% of Class I GIC, 10% of Class I composite, and 10% of Class II composite restorations, along with 20% of Class II GIC restorations, shifted to score 2. However, no restoration was rated below 2 at any point in time. Statistical analysis revealed no significant differences among the groups at baseline, three months, six months, or 12 months (p > 0.05) (Appendix E).

Proximal Contacts

At baseline, three months, and six months, Class II restorations, irrespective of the restorative material (GIC or composite), showed 100% success, and differences between groups were not statistically significant (p = 1.000). At the 12-month recall, in the Class II GIC restorations, four shifted to score 2, and in the composite group, two moved to score 2. There was no statistically significant difference between the two materials at 12 months (Appendix F).

Color Match

At baseline and after three months, all restorations in the four groups were scored as clinically excellent/very good (score 1) with no differences between groups (p = 1.000). At six months, all Class I restorations continued to receive a score of 1. In contrast, in Class II cavities, two GIC restorations scored 2 (clinically sound), while one composite restoration scored 2. These differences were not statistically significant (p = 0.265). At 12 months, three from Class I GIC and two from Class I composite scored 2. In Class II cavities, five of the GIC and four of the composite restorations scored 2. Importantly, no restorations in any group were rated as score 3 or 5. Thus, all restorations remained clinically acceptable at 12 months, with no statistically significant differences among the groups (p = 0.532) (Appendix G).

Wear analysis

Volumetric Change

**Figure 2:**
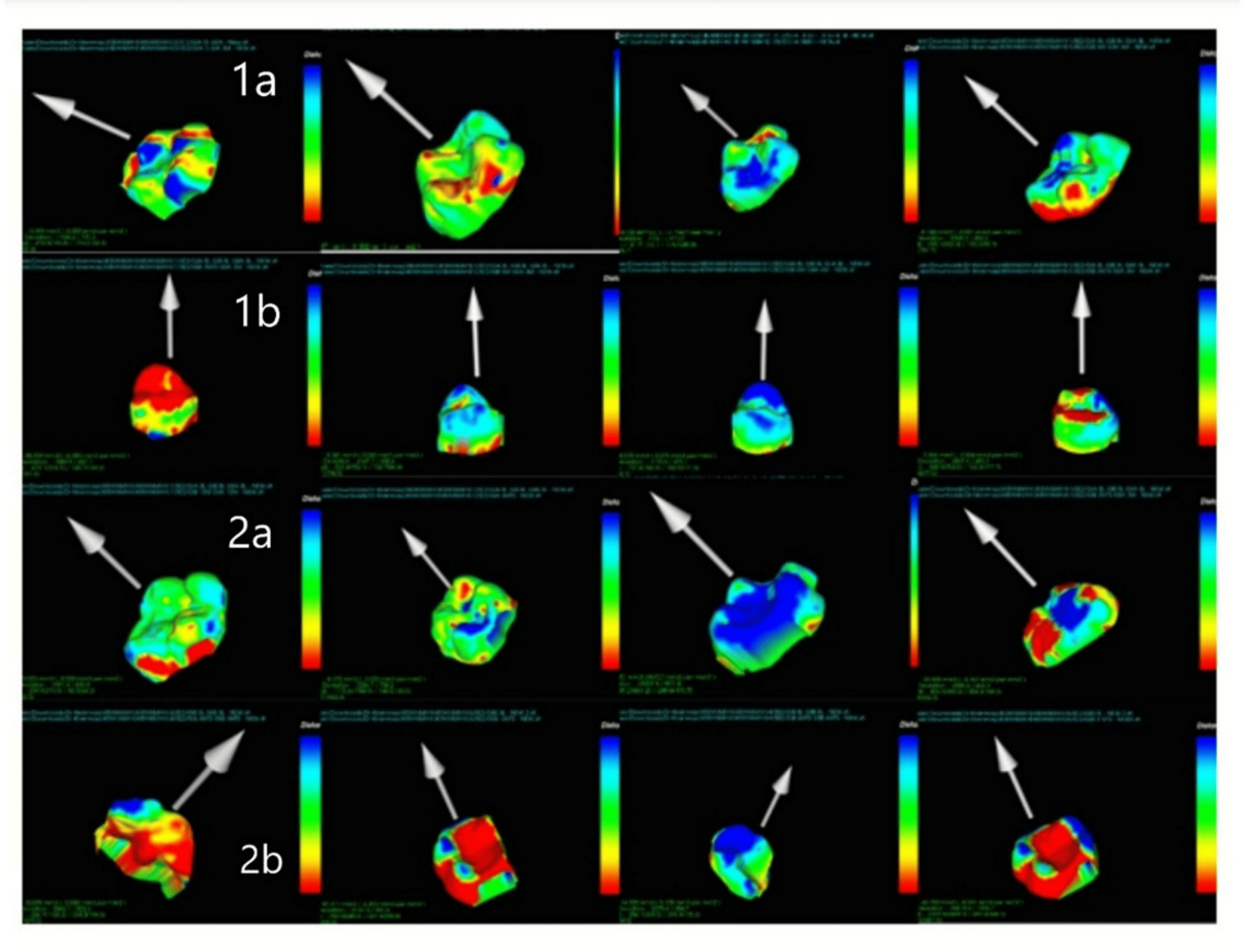
3D color deviation map representing volumetric changes analysis using the Geomagic Control X (3D Systems) and WearCompare software. The baseline (reference) and follow-up period (test) STL files were superimposed using best-fit alignment to assess surface deviations in Groups 1a, 1b, 2a, and 2b. The color scale (in microns) indicates the magnitude and direction of surface change, where green represents minimal deviation, blue shades indicate areas of material gain or positive deviation, and red to yellow regions correspond to material loss or negative deviation STL: standard tessellation language

Comparison of volumetric analysis at various time intervals among the study groups (Table 4) demonstrated mean changes over 12 months, with surface deviations ranging from -479.1 µm to +630.8 µm. In Group 1a, a progressive decrease from baseline was observed at three months (-0.97 ± 1.82), six months (-1.56 ± 2.45), and 12 months (-4.74 ± 6.69), with a statistically significant difference over time (p = 0.048). Group 2a showed minimal volume loss throughout the study period, with mean changes of -0.38 ± 3.03, -0.04 ± 4.73, and +1.18 ± 7.23 at three, six, and 12 months, respectively, which were not statistically significant (p = 0.31). In Group 1b, a consistent decline was recorded from baseline to 12 months (-0.83 ± 1.47, -2.52 ± 3.61, and -4.32 ± 6.22), indicating a significant time-dependent change in volume loss (p = 0.015). Similarly, Group 2b exhibited a progressive, statistically significant reduction from baseline (-1.52 ± 2.53) to six months (-2.64 ± 2.78) and 12 months (-4.20 ± 3.68), indicating a significant loss in volume across all groups (p = 0.002). Overall, Groups 1a, 1b, and 2b demonstrated significant volume loss except for Group 2a class II GIC restorations (Table 5) (Figure 2).

**Table 5:** Comparison of volumetric analysis at various intervals among the study groups Repeated-measures ANOVA was performed, with a p-value of <0.05 considered statistically significant. * means statistically significant. NS: not significant; ANOVA: analysis of variance

Groups	Baseline-3 months (mean (SD))	Baseline-6 months (mean (SD))	Baseline-12 months (mean (SD))	F-value	P-value
Group 1a	-0.974 (1.82)	-1.56 (2.45)	-4.74 (6.69)	3.61	0.048*
Group 2a	-0.3853 (3.03)	-0.044 (4.73)	1.175 (7.23)	1.25	0.31 (NS)
Group 1b	-0.833 (1.47)	-2.518 (3.61)	-4.318 (6.22)	5.31	0.015*
Group 2b	-1.52 (2.53)	-2.64 (2.78)	-4.2 (3.68)	8.74	0.002*

3D Comparison

**Figure 3:**
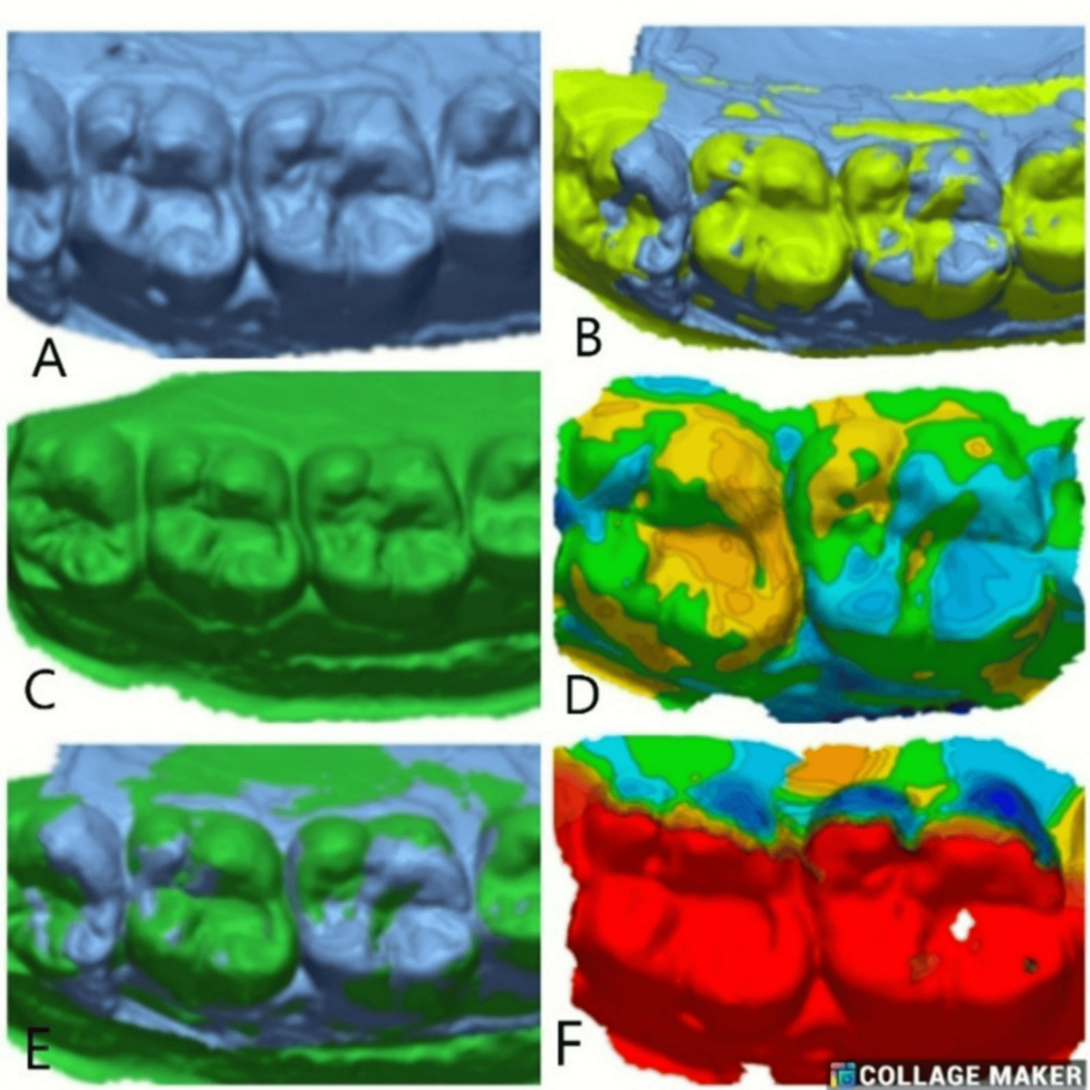
(A) Best-fit alignment comparison of baseline scan for Class I GIC restorations (Group 1a) in the left first and second molars. (B) 3D comparison at three months follow-up after superimposition. (C) Best-fit alignment at six months. (D) 3D comparison at six months follow-up. (E) Best-fit alignment at 12 months follow-up. (F) 3D comparison at 12 months

**Figure 4:**
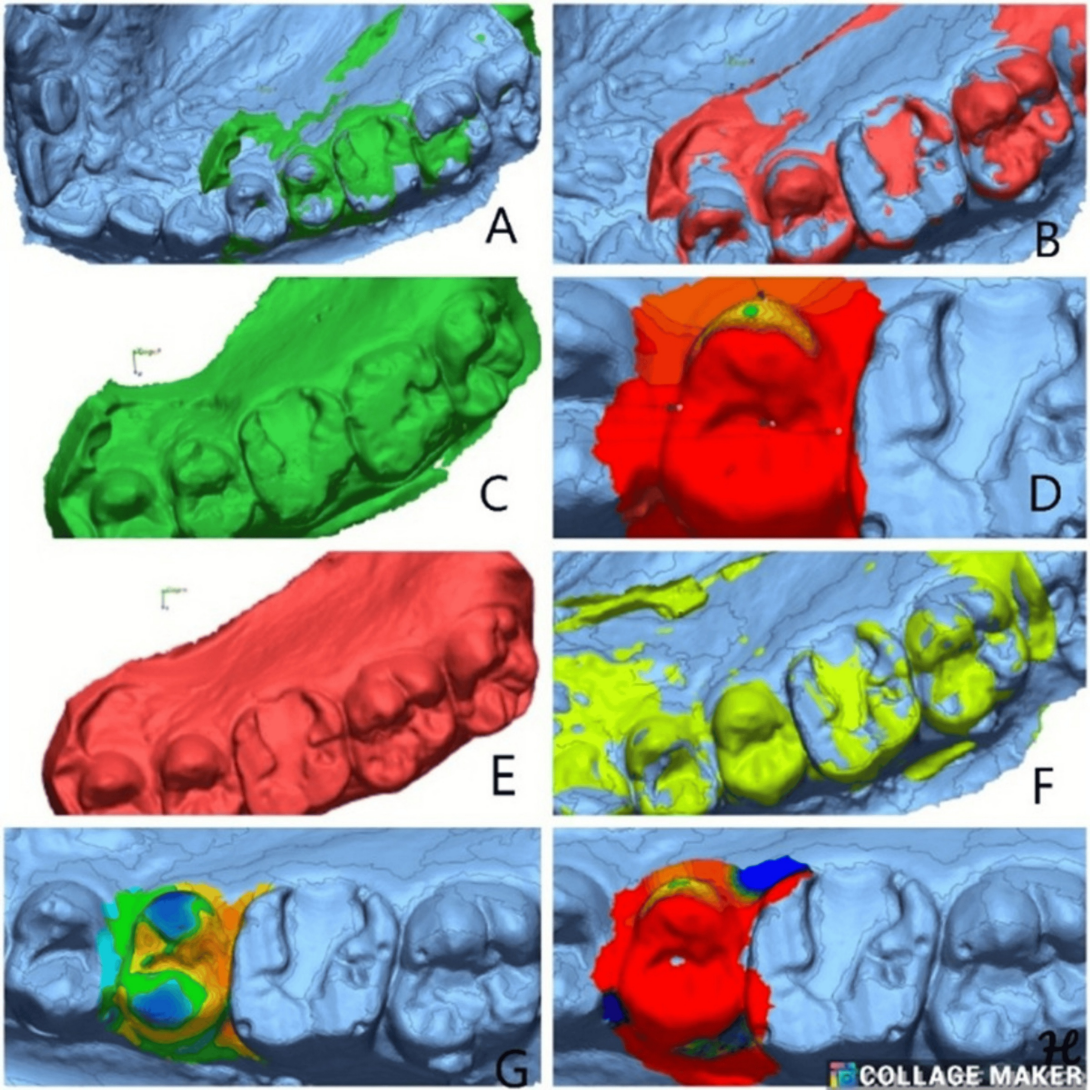
(A) Best-fit alignment comparison of baseline scan for Class I composite restorations (Group 1b) in the right second premolar. (B) 3D comparison for treated tooth after superimposition. (C) Best-fit alignment at three months follow-up. (D) 3D comparison at three months. (E) Best-fit alignment at six months. (F) Best-fit alignment at 12 months follow-up. (G) 3D comparison at six months follow-up. (H) 3D comparison at 12 months

**Figure 5:**
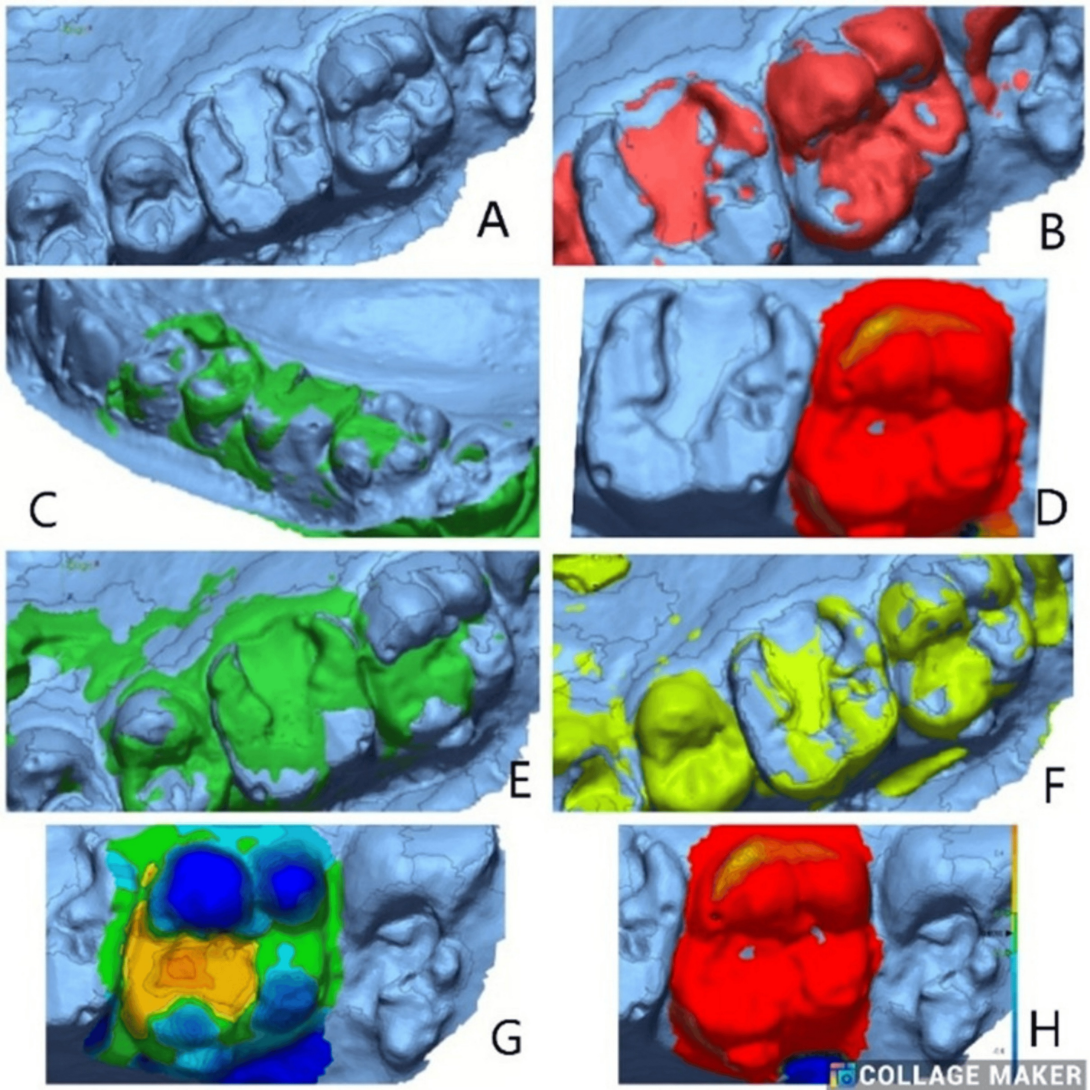
(A) Best-fit alignment comparison of baseline scan for Class II GIC restorations (Group 2a) in the correct second molar. (B) 3D comparison for treated tooth after superimposition. (C) Best-fit alignment at three months follow-up. (D) 3D comparison at three months. (E) Best-fit alignment at six months. (F) Best-fit alignment at 12 months follow-up. (G) 3D comparison at six months follow-up. (H) 3D comparison at 12 months

**Figure 6:**
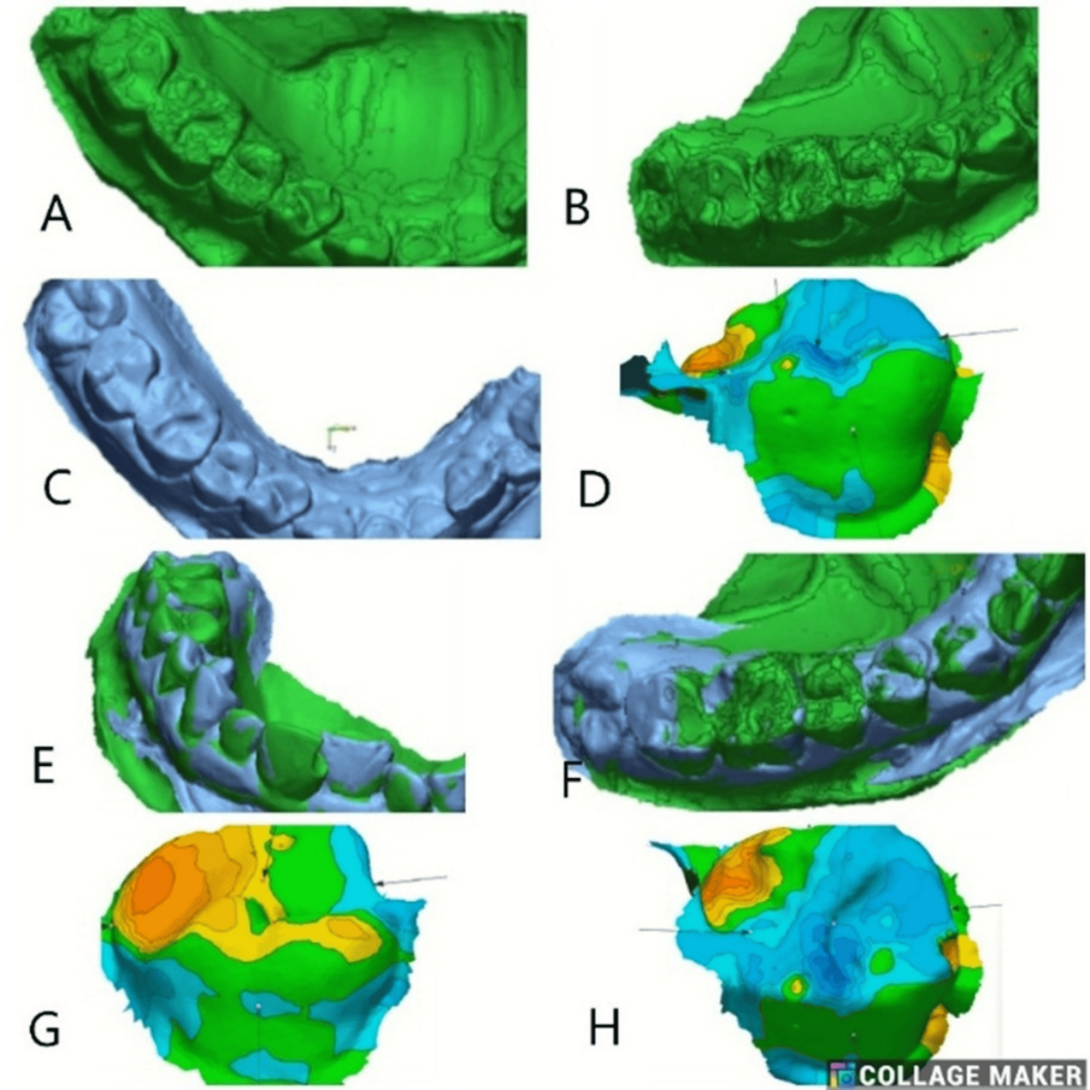
(A) Best-fit alignment comparison of baseline scan for Class II composite (Group 2b) restorations in the left first molar. (B) 3D comparison for treated tooth after superimposition. (C) Best-fit alignment at three months follow-up. (D) 3D comparison at three months. (E) Best-fit alignment at six months. (F) 3D comparison at six months follow-up. (G) Best-fit alignment at 12 months follow-up. (H) 3D comparison at 12 months

Mean values across the four groups, 1a (Figure 3), 1b (Figure 4), 2a (Figure 5), and 2b (Figure 6), at different time intervals showed variations in outcomes over the study period (Table 6). At baseline, statistically significant differences were observed among the groups (p = 0.045), with Group 1b showing the highest mean value (0.16191 ± 0.3326) and Group 2b the lowest (−0.22412 ± 0.3552). At three months, the intergroup differences were not statistically significant (p = 0.363), suggesting relative similarity among the groups. By six months, significant differences re-emerged (p = 0.03), with Group 2b demonstrating the highest mean value (0.75677 ± 0.7218) compared to the other groups. At 12 months, the differences among the groups became more pronounced and statistically significant (p = 0.006), with Group 2b again exhibiting the highest mean (1.46378 ± 1.2050) and Group 1a showing a negative mean change (−0.23486 ± 0.6109). Overall, these findings indicate that significant intergroup variations were present at baseline, six months, and 12 months, while no significant differences were observed at three months. 

**Table 6:** Comparison of volumetric analysis among the study groups NS: not significant

Intervals	Groups	Mean	Std. deviation	F-value	P-value
Baseline and 3 months	Group 1a	-0.9736	1.82	0.283	0.837 (NS)
	Group 2a	-0.3853	3.03		
	Group 1b	-0.8334	1.47		
	Group 2b	-1.5162	2.53		
Baseline and 6 months	Group 1a	-1.5650	2.45	0.854	0.481 (NS)
	Group 2a	-0.0441	4.73		
	Group 1b	-2.5175	3.61		
	Group 2b	-2.6429	2.78		
Baseline and 12 months	Group 1a	-4.7427	6.69	1.577	0.228 (NS)
	Group 2a	1.1750	7.23		
	Group 1b	-4.3182	6.22		
	Group 2b	-4.1996	3.62		

Intragroup comparisons across time intervals revealed that Group 1a showed minor fluctuations in mean values (Table 7), with no statistically significant difference observed (p = 0.107). Similarly, Group 2a demonstrated a gradual increase from baseline (−0.008 ± 0.06) to six months (0.254 ± 0.693), followed by stabilization at 12 months (0.167 ± 0.804), but the difference did not reach statistical significance (p = 0.54). Group 1b also exhibited an increase from baseline (0.162 ± 0.33) to 12 months (0.816 ± 1.40), though this was not statistically significant (p = 0.139). In contrast, Group 2b demonstrated a consistent and marked increase in mean values from baseline (−0.224 ± 0.36) to 12 months (1.46 ± 1.20), which was statistically significant (p < 0.001). Overall, these findings indicate an essential difference (Table 8).

**Table 7:** 3D comparison among the study groups One-way ANOVA was performed, and a p-value of <0.05 was considered statistically significant. * means statistically significant. ANOVA: analysis of variance

Intervals	Groups	Mean	Std. deviation	F-value	P-value
Baseline	Group 1a	0.03815	0.0360	3.29	0.045*
	Group 2a	-0.00752	0.0625		
	Group 1b	0.16191	0.3326		
	Group 2b	-0.22412	0.3552		
3 months	Group 1a	0.08776	0.0775	1.13	0.363
	Group 2a	0.07035	0.1356		
	Group 1b	0.42445	0.5902		
	Group 2b	0.00163	0.5503		
6 months	Group 1a	0.01878	0.2513	3.79	0.03*
	Group 2a	0.25357	0.6927		
	Group 1b	0.61886	1.1726		
	Group 2b	0.75677	0.7218		
12 months	Group 1a	-0.23486	0.6109	5.6	0.006*
	Group 2a	0.16703	0.8037		
	Group 1b	0.81619	1.4136		
	Group 2b	1.46378	1.2050		

**Table 8:** 3D Comparison at various intervals among the study groups Repeated-measures ANOVA was used, with a p-value of <0.05 considered statistically significant. * means statistically significant. NS: not significant; ANOVA: analysis of variance

Groups	Baseline (mean (SD))	3 months (mean (SD))	6 months (mean (SD))	12 months (mean (SD))	F-value	P-value
Group 1a	0.0382 (0.04)	0.0878 (0.08)	0.001 (0.25)	-0.235 (0.6)	2.24	0.107 (NS)
Group 2a	-0.008 (0.06)	0.0703 (0.136)	0.254 (0.693)	0.167 (0.804)	0.732	0.54 (NS)
Group 1b	0.162 (0.33)	0.424 (0.59)	0.619 (1.17)	0.816 (1.4)	0.78	0.139 (NS)
Group 2b	-0.224 (0.36)	0.002 (0.55)	0.757 (0.72)	1.46 (1.2)	14.8	<0.001*

## Discussion

Wear is a significant issue for dental tissues subjected to abrasion, attrition, and erosion processes [9], as well as for dental materials that must be sufficiently wear-resistant without being prejudicial to the opposing teeth. Wear is a common and critical issue for dental materials and dental tissues. They are continuously subjected to occlusal masticatory forces, which result in abrasion, erosion, and attrition; hence, wear is a significant clinical finding. Though it is a vital aspect to resolve, there is no standard method for measuring wear. Various techniques and approaches, including in vitro studies and clinical scanning methods, were employed to measure and calculate wear [10].

Many studies have included tooth wear assessment in the literature, which can be measured using qualitative and quantitative methods. Qualitative methods are subjective and lack precision, accuracy, and reproducibility. In vitro studies are easy to perform, economical, and time-efficient, but they encounter numerous challenges in mimicking the complexity of the oral environment. The assessment of clinical wear through in vitro studies remains controversial, as there is no established standardization of in vitro test conditions. Those methods can be considered semi-quantitative [11]; they do not allow for an accurate wear quantification and tend to underestimate the amount of substance loss. In the literature, various methods are described for evaluating the clinical wear of teeth and dental materials. Many authors measured wear qualitatively using visual assessments and various indices [12]. Some authors measured wear using semi-quantitative methods, such as indirect methods using casts and comparing them with standard models. However, these methods cannot accurately quantify wear, leading to an underestimation of the actual amount of substance loss.

The quality of the scanning procedure is influenced by several parameters, including the width of the laser spot/stylus size, the resolution in the three axes (x, y, z), the scanning step, the sample angulation, the depth range, and the material's optical properties. The obtained cloud of points is subsequently processed using mathematical models to extrapolate the entire surface. At each evaluation time, the digital reconstructions obtained are superimposed using the metrology software [13].

The use of intraoral scanners is rapidly advancing across various fields of dentistry. They were already regularly used to make diagnostic cast impressions, transfer records, and record measurements for a prosthesis. Recently, they have been introduced to measure the wear of dental tissues and restorative materials. Some studies suggest that 3D software and the scanning method used to measure wear are accurate in clinical settings. This may be a complex method for assessing occlusal tooth/restorative material wear through serial 3D surface model superimpositions. There is a need for a study in the literature that addresses this issue [14]. Measuring the wear of restorative materials requires comparing a baseline 3D scan of the restoration with follow-up scans taken at later points in time. The International Organization for Standardization (ISO) recommended that Geomagic Control X facilitates this process with its robust comparison tools.

The models were scanned three-dimensionally, and these scans are saved as STL files. The STL files from the baseline and follow-up scans are imported into Geomagic Control X [15]. The software's "best-fit" algorithm aligns the follow-up scan (the "measured data") with the baseline scan (the "reference data"). This accurately superimposes the two datasets, allowing for direct comparison. A "3D compare" tool generates a color-coded deviation map. This map visually shows the differences between the reference and measured surfaces [16]. The color scale can be adjusted to represent specific levels of wear visually. For example, a shift from blue to red might indicate an increase in material loss, while green indicates no change. The software calculated parameters, such as maximum occlusal height loss and mean volume loss, to assess the long-term clinical performance of different restorative materials [17]. The "3D Compare Tool" of the software was used to generate color maps for the quantitative evaluation of the deviations at the external surfaces. Overcontoured areas are indicated in red color, and undercontoured areas are indicated in blue color, with maximum/minimum deviation values set at 100 µm. The tolerance range was set to 10 µm and indicated in green [18].

The findings of the present study demonstrated notable variations among the four groups (1a, 2a, 1b, and 2b) across different evaluation periods, reflecting time-dependent changes in the measured parameter. Statistically significant intergroup differences were evident at baseline, six months, and 12 months, whereas no significant differences were observed at three months. This pattern suggests a dynamic response of the tested variables over time, influenced by both the intrinsic properties of the groups and the duration of exposure or intervention.

At baseline, significant variation was observed among groups, with the Class I composite group exhibiting the highest mean value and the Class II composite group the lowest. This variation could be attributed to inherent differences in the basic cavity design. However, the lack of statistical significance at three months suggests a period of stabilization, possibly reflecting the material's initial adaptation to the different cavity configurations. The significant differences at six and 12 months indicate a progressive increase in mean values in Group 2b, suggesting that the material is undergoing wear over time. In contrast, the negative mean change in Group 1a at 12 months suggests potential material loss or wear, highlighting variability in long-term performance or response.

Ideally, a restoration should have wear resistance similar to that of enamel. As the attrition of the enamel cusp totals up to 68 pm a year (this is in accordance with the study of Lambrechts et al. [5]), all materials overstepping this limit are contraindicated as posterior restorative.

The intervention group in the study used GlasIonomer FX Ultra, a truly self-adhesive, bulk-filling glass ionomer restorative. It outperforms conventional restoratives in its class with its unique new formulation, providing superior esthetics, translucency, lasting caries resistance, and stability. GlasIonomer FX Ultra features an optimally balanced formulation with high initial reactivity and rapid maturation. This virtually equalizes the refractive indices of the glass core and the matrix, providing a remarkably stable, strong, translucent, and fluoride-rich restoration. It has improved mechanical strength (higher compressive, diametral tensile, or flexural strength) compared with conventional GIC. It was reported to be strong and durable, consistently enduring intraoral forces due to its superior mechanical properties. A few studies by Doumit et al. [19] show that even reinforced GICs (Shofu RX Ease) tend to exhibit higher wear than resin composites under certain conditions. Until now, no study has tested and compared the wear rates and clinical performance of GlasIonomer FX Ultra with Tetric EvoCeram. In the present study, although the difference was not statistically significant, GIC demonstrated good clinical performance up to 12 months, but exhibited a slightly lower performance rate than bulk-fill composite.

The Tetric EvoCeram composite used in the study is a light-curing, radiopaque nanohybrid bulk-fill composite based on the latest technology for direct restorative therapy. It is a new universal material from Ivoclar Vivadent with high viscosity, designed for use as a posterior direct restorative material. It was ensured that the composite was adequately cured to achieve optimal polymer network conversion. It enhances the composite's mechanical properties, enabling it to withstand wear in the oral cavity [20]. Hence, the curing time was followed according to the manufacturer's instructions in the study.

According to the literature, adequate energy needed to cure a 2 mm increment of composite resin typically requires a minimum of 16-24 J/cm² of radiant exposure (energy density). Since the total energy delivered to the composites was 1000 mW/cm^2^, the curing time was set to 16-24 seconds. It agrees with the recommendations found in the literature. The increased wear resistance of the Tetric EvoCeram composite resin is likely due to the influence of improved conventional properties on filler retention, rather than the wear resistance of the pure matrix itself, which shows wear resistance at contact points. Therefore, bulk-fill composites can be recommended for the restoration of Class II cavity preparations.

The greater the dimensions of restorations, the larger the surface area exposed to masticatory stresses, and the faster the restoration will undergo material loss [21]. In addition, beveling of the occlusal margin increases the dimension of the restoration's contact in occlusion, inviting increased wear and fracture [22]. Therefore, increased surface area of restoration, chipping of overhangs and localized marginal fractures in and around the bevels, physiologic attrition wear at occlusal contact areas, and diet-induced abrasive wear at contact-free occlusal areas, all together might have contributed to increased volume loss of restorative surfaces versus enamel observed in the study by Komireddy et al. [23].

The progressive divergence among the groups over 12 months underscores the importance of long-term evaluation when assessing the durability or persistence of an intervention. Short-term observations alone may fail to capture delayed or compounding effects that become evident only after extended periods. The present results align with earlier studies reporting time-dependent changes in wear or volume loss of restorative materials, emphasizing that both the nature of the material or intervention and the duration of exposure play a significant role in determining outcomes [24].

The study's findings indicate that multiple factors are associated with the wear of direct restorative materials, including chewing cycles, forces exerted during mastication, the abrasive medium used for brushing, eating habits, and the type of food consumed. Overall, the results of this study indicate that while transient uniformity may occur during the mid-term period, significant divergence in group responses becomes evident over time. This emphasizes the necessity of longitudinal monitoring and selecting interventions that show stable or improving trends over extended durations. Even with a composite like Tetric EvoCeram, good technique, bonding, and occlusal adjustment are essential for achieving a favorable wear outcome.

Clinically, these findings emphasize the importance of assessing long-term stability rather than relying solely on short-term outcomes when selecting a treatment, material, or intervention. The present clinical study evaluated and compared the performance of GIC and composite resin restorations in Class I and Class II cavities over 12 months, focusing on multiple parameters, including fracture, retention, marginal adaptation, secondary caries, color match, postoperative sensitivity, and proximal contact integrity. Overall, both restorative materials exhibited excellent clinical performance up to 12 months, with only minor, non-significant deteriorations observed toward the end of the study period.

Fracture resistance and retention

In the study, both GIC and composite restorations exhibited reliable retention and resistance to fracture for up to six months, consistent with previous studies reporting high success rates for both materials in posterior teeth [25]. Class II GIC restorations exhibited a slight decline at 12 months, although this was not statistically significant. It may be stated that larger cavity size and higher occlusal loading in proximal areas may compromise mechanical integrity and show the inherent brittleness and lower flexural strength of GIC compared to resin-based composites.

Marginal adaptation and integrity

Up to six months, GIC and composite restorations maintained 100% marginal integrity, reflecting optimal initial bonding and adaptation. At 12 months, minor marginal changes emerged in both materials, particularly in Class II cavities. These changes could be attributed to polymerization shrinkage stresses in composites and gradual erosion or wear at the margins in GICs. Despite these minor changes, none of the restorations exhibited gross failure and remained within clinically acceptable levels. Results have shown that proper isolation can reinforce the long-term stability of both materials.

Secondary caries

At 12 months, no cases of secondary caries were recorded in either restoration group, possibly due to their marginal sealing ability. The fluoride-releasing properties of GIC likely contributed to its caries-preventive potential, while adequate adhesive bonding and surface finish helped composites resist marginal leakage. The absence of statistically significant differences between groups supports the notion that both materials, when properly maintained, provide sufficient protection against recurrent decay within the first year of service.

Cavo-surface margin discoloration

Marginal discoloration was minimal and not statistically significant at any interval, though slightly higher percentages were observed in Class II restorations at 12 months. This can be attributed to increased plaque retention in proximal areas and potential microleakage under masticatory stress [26]. The greater discoloration tendency in composites compared to GICs may relate to the resin matrix's susceptibility to surface staining from dietary pigments and water sorption over time. Nonetheless, all restorations retained acceptable esthetic scores (≤2), confirming their clinical adequacy.

Postoperative sensitivity

No postoperative sensitivity was reported up to six months, and only minor, non-significant increases occurred at 12 months. This favorable outcome reflects the adequate sealing and adaptation of both restorative materials, which minimize microleakage and fluid movement across dentinal tubules. Slightly higher sensitivity in Class II composites at 12 months may be related to polymerization stress or marginal gap formation in deeper proximal boxes.

Gross fracture and surface integrity

Minor deterioration and surface wear were observed in a small number of restorations at 12 months, particularly in GICs placed in Class II cavities. These findings are consistent with previous reports highlighting GIC's lower resistance to flexural and tensile stresses [27]. Composites showed slightly better long-term stability, possibly due to their higher filler loading and superior elastic modulus, which better accommodates occlusal stresses. However, the absence of catastrophic fractures or restoration loss reinforces the satisfactory performance of both materials in moderate stress-bearing areas over the short term.

Proximal contacts

Proximal contact integrity for Class II cavities, especially GIC restorations, showed a significant reduction in contact tightness at 12 months. This may be due to material wear or slight deformation under occlusal load, resulting in a marginal gap. The findings correlated with other studies, showing more frequent proximal wear and contact loss in GIC restorations compared to resin composites in posterior teeth [28].

Color match

Both materials maintained excellent color stability for the first six months, with minor deterioration observed at 12 months, especially in Class II restorations. Although these changes were not statistically significant, they may be related to surface roughness, matrix degradation, or dietary staining. Composites generally showed better esthetic stability than GICs, consistent with their superior optical properties and polishability [29].

Overall interpretation

The present findings indicate that both GIC and composite resins achieve high clinical success for Class I and II restorations for up to 12 months, with no statistically significant differences between the materials. However, a trend toward reduced performance in GIC Class II restorations suggests that cavity size and stress concentration influence restoration longevity. These results emphasize the importance of case selection; GICs may be better suited for low-stress Class I cavities or as atraumatic restorative treatment (ART) materials. At the same time, composites may be preferable in areas subjected to higher occlusal loads and esthetic demands [30].

Clinical implications

Clinicians can confidently use both materials for small- to moderate-sized posterior restorations, provided appropriate placement and finishing protocols are followed. Regular monitoring beyond 12 months is crucial to evaluate long-term performance, especially for GICs in high-stress regions. Future studies with more extended follow-up periods and larger sample sizes could further elucidate the material-dependent degradation patterns observed over time. 

Strengths and limitations

The study has several important strengths that enhance its methodological rigor and clinical relevance. These include the randomized clinical design combined with parallel in vitro and in vivo assessments, allowing controlled laboratory wear findings to be interpreted alongside clinical performance. The use of standardized restorative protocols performed by a single operator minimized procedural variability, while the incorporation of objective 3D volumetric wear analysis provided a quantitative assessment beyond conventional visual evaluation. In addition, the application of the validated revised FDI criteria, together with examiner calibration and high inter-examiner agreement, strengthens the reliability and consistency of the clinical outcomes. The authors appropriately acknowledge the 12-month follow-up period as a primary limitation, as this duration is insufficient to fully assess long-term wear behavior and restoration longevity. Additional limitations that warrant consideration include the relatively small sample size and single-center study design, which may restrict the generalizability of the findings to broader clinical populations and practice settings. Although the use of a single operator enhanced procedural standardization, it may also introduce operator-dependent effects, potentially limiting external validity. Overall, while the strengths and limitations of the study are largely accurately described, a more explicit discussion of these methodological and clinical constraints provides a more balanced interpretation of the results and helps contextualize their applicability in routine clinical practice.

## Conclusions

Within the limitations of this study, both GIC and composite resins demonstrated excellent clinical success for Class I and II restorations up to 12 months. Both materials remain viable options for posterior restorations, provided appropriate case selection and placement techniques are followed. If the primary concern is wear resistance under occlusal load (especially in posterior, high-stress areas), Tetric EvoCeram has the more substantial evidence. FX Ultra might be acceptable when GIC advantages (fluoride release, chemical adhesion, and a more straightforward technique) outweigh the disadvantages (moderate loads and limited fluoride release) and when loads are moderate. However, there is a potential for higher wear or shorter durability in high-load posterior applications compared to the composite. GlasIonomer FX Ultra, marketed as high-strength/ultra-GIC, likely has greater wear resistance than a modern resin composite but less than that of nanohybrid bulk-fill composites.

## Figures and Tables

**Figure 7:**
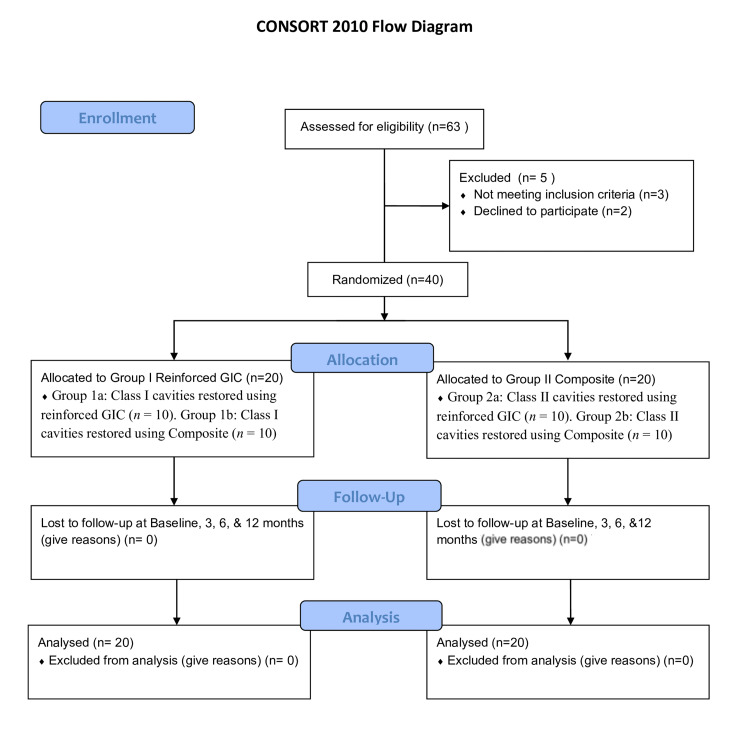
Consort flow diagram for the study GIC: glass ionomer cement

## Appendices

Appendix A

Appendix B

**Table 9:** Secondary caries GIC: glass ionomer cement; NS: not significant

Intervals	Groups	1	2	3	4	5	P-value
Baseline	Class I GIC	10 (100)	0 (0)	0 (0)	0 (0)	0 (0)	1.000 (NS)
	Class I composite	10 (100)	0 (0)	0 (0)	0 (0)	0 (0)	
	Class II GIC	10 (100)	0 (0)	0 (0)	0 (0)	0 (0)	
	Class II composite	10 (100)	0 (0)	0 (0)	0 (0)	0 (0)	
3 months	Class I GIC	10 (100)	0 (0)	0 (0)	0 (0)	0 (0)	1.000 (NS)
	Class I composite	10 (100)	0 (0)	0 (0)	0 (0)	0 (0)	
	Class II GIC	10 (100)	0 (0)	0 (0)	0 (0)	0 (0)	
	Class II composite	10 (100)	0 (0)	0 (0)	0 (0)	0 (0)	
6 months	Class I GIC	10 (100)	0 (0)	0 (0)	0 (0)	0 (0)	1.000 (NS)
	Class I composite	10 (100)	0 (0)	0 (0)	0 (0)	0 (0)	
	Class II GIC	10 (100)	0 (0)	0 (0)	0 (0)	0 (0)	
	Class II composite	10 (100)	0 (0)	0 (0)	0 (0)	0 (0)	
12 months	Class I GIC	10 (100)	0 (0)	0 (0)	0 (0)	0 (0)	0.473 (NS)
	Class I composite	9 (90)	1 (10)	0 (0)	0 (0)	0 (0)	
	Class II GIC	8 (80)	2 (20)	0 (0)	0 (0)	0 (0)	
	Class II composite	8 (80)	2 (20)	0 (0)	0 (0)	0 (0)	

Appendix C

**Table 10:** Cavo-surface margin discoloration GIC: glass ionomer cement; NS: not significant

Intervals	Groups	1	2	3	4	5	P-value
Baseline	Class I GIC	10 (100)	0 (0)	0 (0)	0 (0)	0 (0)	1.000 (NS)
	Class I composite	10 (100)	0 (0)	0 (0)	0 (0)	0 (0)	
	Class II GIC	10 (100)	0 (0)	0 (0)	0 (0)	0 (0)	
	Class II composite	10 (100)	0 (0)	0 (0)	0 (0)	0 (0)	
3 months	Class I GIC	10 (100)	0 (0)	0 (0)	0 (0)	0 (0)	1.000 (NS)
	Class I composite	10 (100)	0 (0)	0 (0)	0 (0)	0 (0)	
	Class II GIC	10 (100)	0 (0)	0 (0)	0 (0)	0 (0)	
	Class II composite	10 (100)	0 (0)	0 (0)	0 (0)	0 (0)	
6 months	Class I GIC	10 (100)	0 (0)	0 (0)	0 (0)	0 (0)	1.000 (NS)
	Class I composite	10 (100)	0 (0)	0 (0)	0 (0)	0 (0)	
	Class II GIC	10 (100)	0 (0)	0 (0)	0 (0)	0 (0)	
	Class II composite	10 (100)	0 (0)	0 (0)	0 (0)	0 (0)	
12 months	Class I GIC	9 (90)	1 (10)	0 (0)	0 (0)	0 (0)	0.446 (NS)
	Class I composite	8 (80)	2 (20)	0 (0)	0 (0)	0 (0)	
	Class II GIC	6 (60)	4 (40)	0 (0)	0 (0)	0 (0)	
	Class II composite	7 (70)1	3 (30)	0 (0)	0 (0)	0 (0)	

Appendix D

**Table 11:** Postoperative sensitivity GIC: glass ionomer cement; NS: not significant

Intervals	Groups	1	2	3	4	5	P-value
Baseline	Class I GIC	10 (100)	0 (0)	0 (0)	0 (0)	0 (0)	1.000 (NS)
	Class I composite	10 (100)	0 (0)	0 (0)	0 (0)	0 (0)	
	Class II GIC	10 (100)	0 (0)	0 (0)	0 (0)	0 (0)	
	Class II composite	10 (100)	0 (0)	0 (0)	0 (0)	0 (0)	
3 months	Class I GIC	10 (100)	0 (0)	0 (0)	0 (0)	0 (0)	1.000 (NS)
	Class I composite	10 (100)	0 (0)	0 (0)	0 (0)	0 (0)	
	Class II GIC	10 (100)	0 (0)	0 (0)	0 (0)	0 (0)	
	Class II composite	10 (100)	0 (0)	0 (0)	0 (0)	0 (0)	
6 months	Class I GIC	10 (100)	0 (0)	0 (0)	0 (0)	0 (0)	1.000 (NS)
	Class I composite	10 (100)	0 (0)	0 (0)	0 (0)	0 (0)	
	Class II GIC	10 (100)	0 (0)	0 (0)	0 (0)	0 (0)	
	Class II composite	10 (100)	0 (0)	0 (0)	0 (0)	0 (0)	
12 months	Class I GIC	9 (90)	1 (10)	0 (0)	0 (0)	0 (0)	0.741 (NS)
	Class I composite	8 (80)	2 (20)	0 (0)	0 (0)	0 (0)	
	Class II GIC	8 (80)	2 (20)	0 (0)	0 (0)	0 (0)	
	Class II composite	7 (70)	3 (30)	0 (0)	0 (0)	0 (0)	

Appendix E

**Table 12:** Gross fracture GIC: glass ionomer cement; NS: not significant

Intervals	Groups	1	2	3	4	5	P-value
Baseline	Class I GIC	10 (100)	0 (0)	0 (0)	0 (0)	0 (0)	1.000 (NS)
	Class I composite	10 (100)	0 (0)	0 (0)	0 (0)	0 (0)	
	Class II GIC	10 (100)	0 (0)	0 (0)	0 (0)	0 (0)	
	Class II composite	10 (100)	0 (0)	0 (0)	0 (0)	0 (0)	
3 months	Class I GIC	10 (100)	0 (0)	0 (0)	0 (0)	0 (0)	1.000 (NS)
	Class I composite	10 (100)	0 (0)	0 (0)	0 (0)	0 (0)	
	Class II GIC	10 (100)	0 (0)	0 (0)	0 (0)	0 (0)	
	Class II composite	10 (100)	0 (0)	0 (0)	0 (0)	0 (0)	
6 months	Class I GIC	10 (100)	0 (0)	0 (0)	0 (0)	0 (0)	1.000 (NS)
	Class I composite	10 (100)	0 (0)	0 (0)	0 (0)	0 (0)	
	Class II GIC	10 (100)	0 (0)	0 (0)	0 (0)	0 (0)	
	Class II composite	10 (100)	0 (0)	0 (0)	0 (0)	0 (0)	
12 months	Class I GIC	9 (90)	1 (10)	0 (0)	0 (0)	0 (0)	0.877 (NS)
	Class I composite	9 (90)	1 (10)	0 (0)	0 (0)	0 (0)	
	Class II GIC	8 (80)	2 (20)	0 (0)	0 (0)	0 (0)	
	Class II composite	9 (90)	1 (10)	0 (0)	0 (0)	0 (0)	

Appendix F

**Table 13:** Proximal contact * means a statistically significant difference. GIC: glass ionomer cement; NS: not significant

Intervals	Groups	1	2	3	4	5	P-value
Baseline	Class II GIC	10 (100)	0 (0)	0 (0)	0 (0)	0 (0)	1.000 (NS)
	Class II composite	10 (100)	0 (0)	0 (0)	0 (0)	0 (0)	
3 months	Class II GIC	10 (100)	0 (0)	0 (0)	0 (0)	0 (0)	1.000 (NS)
	Class II composite	10 (100)	0 (0)	0 (0)	0 (0)	0 (0)	
6 months	Class II GIC	10 (100)	0 (0)	0 (0)	0 (0)	0 (0)	1.000 (NS)
	Class II composite	10 (100)	0 (0)	0 (0)	0 (0)	0 (0)	
12 months	Class II GIC	6 (60)	4 (40)	0 (0)	0 (0)	0 (0)	0.035*
	Class II composite	8 (80)	2 (20)	0 (0)	0 (0)	0 (0)	

Appendix G

**Table 14:** Color match GIC: glass ionomer cement; NS: not significant

Intervals	Groups	1	2	3	4	5	P-value
Baseline	Class I GIC	10 (100)	0 (0)	0 (0)	0 (0)	0 (0)	1.000 (NS)
	Class I composite	10 (100)	0 (0)	0 (0)	0 (0)	0 (0)	
	Class II GIC	10 (100)	0 (0)	0 (0)	0 (0)	0 (0)	
	Class II composite	10 (100)	0 (0)	0 (0)	0 (0)	0 (0)	
3 months	Class I GIC	10 (100)	0 (0)	0 (0)	0 (0)	0 (0)	1.000 (NS)
	Class I composite	10 (100)	0 (0)	0 (0)	0 (0)	0 (0)	
	Class II GIC	10 (100)	0 (0)	0 (0)	0 (0)	0 (0)	
	Class II composite	10 (100)	0 (0)	0 (0)	0 (0)	0 (0)	
6 months	Class I GIC	10 (100)	0 (0)	0 (0)	0 (0)	0 (0)	0.265 (NS)
	Class I composite	10 (100)	0 (0)	0 (0)	0 (0)	0 (0)	
	Class II GIC	8 (80)	2 (20)	0 (0)	0 (0)	0 (0)	
	Class II composite	9 (90)	1 (10)	0 (0)	0 (0)	0 (0)	
12 months	Class I GIC	7 (70)	3 (30)	0 (0)	0 (0)	0 (0)	0.532 (NS)
	Class I composite	8 (80)	2 (20)	0 (0)	0 (0)	0 (0)	
	Class II GIC	5 (50)	5 (50)	0 (0)	0 (0)	0 (0)	
	Class II composite	6 (60)	4 (40)	0 (0)	0 (0)	0 (0)	
